# Performative Masculinity: A META-Ethnography of Experiences of Men in Academic and Clinical Nursing

**DOI:** 10.3390/ijerph192214813

**Published:** 2022-11-10

**Authors:** Hsing-Yuan Liu, Hui-Mei Han, Chun-Yen Chao, Hsiu-Fang Chen, Sheau-Ming Wu

**Affiliations:** 1 Department of Nursing, Chang Gung University of Science and Technology, Taoyuan City 33303, Taiwan; 2 Department of Nursing, Linkous Chang Gung Memorial Hospital, Taoyuan City 33303, Taiwan; 3 Department of Nursing, Chang Gung University, Taoyuan City 33303, Taiwan; 4 Department of Nursing, New Taipei Municipal Tu Cheng Hospital, New Taipei 236017, Taiwan; 5 Department of Cosmetic, Chang Gung University of Science and Technology, Taoyuan City 33303, Taiwan

**Keywords:** men in nursing, meta-ethnography, performative masculinity

## Abstract

Gender differences and stereotypes have been widely studied. Performative masculinity or “doing gender” is the adoption of traits considered to be “masculine” to achieve acceptance in society. Performative masculinity, as it relates to career development for men in nursing, could be affected by internal attitudes and career choice, and external influences of culture. There are no critical systematic reviews to describe this process. The aim of this study was to synthesize research on how men in nursing experience masculine identity at work. A systematic literature search and qualitative synthesis using meta-ethnography guided this study. The literature search included studies from the electronic databases of PubMed, CINAHL, Medline, and Airiti Library, published in English or Chinese from 1994 to 2016. Eleven studies met the inclusion criteria. The meta-syntheses identified three themes describing how men in nursing experience masculine identity at work: (1) Nursing could be a gendered-neutral occupation suitable to both genders; (2) performative masculinity; and (3) strategies used when providing bodywork and care. Exploring insights about the tensions of performative masculinity among men in nursing, this study may help to address the challenges encountered in nursing education, practice, and career development. Establishing a male nursing model based on evidence from academic and clinical practice for nursing students and professional nurses could help to meet the increasing demand for nurses.

## 1. Introduction

The English word ‘nurse’ originates from the Latin words ‘nutrire’, which means to nourish, and ‘nutrix’, which means nursing mother. As a verb, it denotes not only the act of attending to the needy, but also the act of breastfeeding a child. As such, the word nurse reflects the unquestionable duty of females [1]. Furthermore, the sexual division of the workplace within medicine has been a logical extension of the male–female role relations in society. The traditional doctor–nurse relationship has been a man–woman relationship. Consciously or unconsciously, nursing work has been associated with femininity and less power or authority.

Nursing is a female-dominated profession, particularly within the less prestigious positions. A survey of 104 countries across different regions reported relatively low percentages of male nurses, including Africa (35%), Eastern Mediterranean Region (21%), Southeast Asia (21%), Western Pacific (19%), Europe (16%), and the Americas (14%) [2]. However, the number of male nurses is increasing in both developing and developed economies worldwide. For example, male registered nurses employed in Japan increased from 22,189 in 2000 to 53,748 in 2010 [3]. In South Africa, the number of male professional nurses had risen from 1841 to 5244, and the number of male trainees had doubled from 785 to 1555 in 2009 [4]. In Taiwan, the proportion has been increasing continuously in recent years (from 0.8% in 2008 to 3.6% in 2021) [5]. Nonetheless, many nursing education programs and clinical care facilities struggle to attract and retain men. Indeed, in 2016 only 9.41% of the students enrolled in Taiwan’s nursing programs were men, and only 2.11% of the nurses practicing in clinics were men [6]. These findings are best summarized by Harding et al.’s [7] report that ‘fewer men are still enrolling in nursing programs, are less likely to graduate and less likely to remain in the nursing workforce postgraduation’.

Several studies have explained the high turnover rate of male nurses as resulting from: (a) the stereotyping of nursing as a “woman’s job” [8]); (b) the stereotyping of male nurses as sexual aggressors leading to patients’ refusal of physical contact during care [9].; and (c) the labeling of male nurses as “sissies,” homosexuals, and perverts [10]. However, stereotyping or gender bias alone cannot explain the complex phenomena and situations men encounter in nursing educational and clinical contexts.

The lack of male nurses in the nursing profession should be viewed as a public health concern. Male representation in nursing is critical, as it not only helps to broaden gender diversity, but also can help reduce nursing shortages and improve the quality of medical services and treatments. Indeed, improving workforce diversity is a priority of professional nursing stakeholders nationally and internationally. It is vital to determine strategies for recruiting, training, and retaining male nursing students [11].

## 2. Background

Men are encouraged to distance themselves from work perceived as feminine to retain the privilege of “hegemonic masculinity” [12], in which a dominant form of masculinity serves as the paragon. Butler [13] described gender as performative in nature; not something we are born with, but something we do (“doing gender”). Other sociologists [14,15] have expanded on performativity and contended that gender and its related roles change with time and are fluid with one’s culture. Although men in nursing experience contradictions and tensions with respect to masculine identity [16], studies on how men negotiate and perform masculinities are limited.

Hegemonic masculinity is embedded in a “historically dynamic view of gender” [17], which influences one’s subjective feeling of power and formation of identity [18]. Doing gender, or negotiating gendered behavior, has been proposed to explain the phenomenon [19,20]. Pitt and Fox [21,22] replaced the term “hegemonic masculinity” with ‘performative masculinity,’ which integrated Bourdieu’s [20,23] concept of “masculine habitus,” West and Zimmerman’s [20] theory of “doing gender”, and Butler’s [24] concept of “performative gender.” The term ‘performative masculinity’ allows masculinity to be described in a socio-cultural context. For example, the nursing profession is not considered a traditional career choice for men [18]. For men to enter nursing requires negotiation of emotions [19,25]. Performative masculinity provides a broader perspective of emotionality in terms of fluidity, contingency, and performativity of gender. In other words, “soft masculinity” coined by Holyoake is one way through which men combat unwanted stereotypes [26]. 

Knowledge of performative masculinity in academic and clinical nursing environments could provide an understanding of challenges men encounter during nursing education and career development. A systematic review using meta-ethnography is needed, which is useful for qualitative synthesis when the research aim is to aggregate findings to develop a conceptual understanding of a phenomenon [27]. A meta-ethnography analyzes and interprets individual qualitative studies to answer larger questions regarding a specific topic. By drawing on analogies and relationships between concepts and metaphors that may be hidden within individual studies [28], a meta-ethnography can illustrate patterns of the mentality of men in nursing may express toward certain ideas and beliefs through their language, as well as individual and group behaviors observed by the researchers. Previous work on hegemonic masculinity has established that the nature of masculinity is hierarchical, relational, and located in specific cultural context [12,17]. As Inhorn and Wentzell [29] argued, theories on masculinity must become more dynamic to account for the constant, embodied, inter-related changes in masculine identities and practices. The authors also indicated that that performative masculinity explains the ongoing, relational, and embodied ways in which male nurses enact masculinity.

Therefore, the aim of this study was to understand the challenges encountered by men in nursing over the course of their career development, specifically in clinical and academic practices. Using meta-ethnography to explore male nurses ‘experiences in academic and clinical settings, this study examined their performative masculinity in different cultural contexts. The findings of this study could generate an explanatory framework from which to develop a model of men in nursing for use in future studies by nursing researchers, educators, and administrators. A model of men in nursing could facilitate nursing education programs and clinical care facilities in attracting and retaining men. Specifically, our objectives were to understand what men in nursing do to exhibit masculinity and to present this information as a basis from which to develop a future model of men in nursing. The following research questions guided this study: (a) Is nursing a gendered profession? and (b) how do men in nursing construct and negotiate performance masculinity?

## 3. Materials and Methods

### 3.1. Research Design

This study used the meta-ethnography approach to qualitative synthesis proposed by Noblit and Hare [28]. The seven-step meta-ethnography approach used for this study included a literature search, assessment of quality, and synthesis. This method was chosen for its strong ability to yield complex, detailed descriptions of a group culture by exploring social phenomena rather than testing hypotheses when interpreting human actions.

### 3.2. Search Method and Outcomes

A computer-based literature search conducted in May 2017 employed the electronic databases of PubMed, CINAHL, ProQuest, and the Taiwan Periodical Literature System to extract full-text studies published from 1994 to 2016 in English or Chinese. The key words included the combinations of male nursing, masculinity, and qualitative research. The first and second authors independently identified relevant articles by reviewing citations, abstracts, and full texts. Discrepancies in the study selections were discussed and the final inclusions were based on consensus. Logically based inclusion criteria for selecting studies were: (a) studies used qualitative research design, and (b) studies focused on masculinity. The exclusion criteria were: (a) full texts not available, (b) non-English or non-Chinese papers.

**Figure 1:**
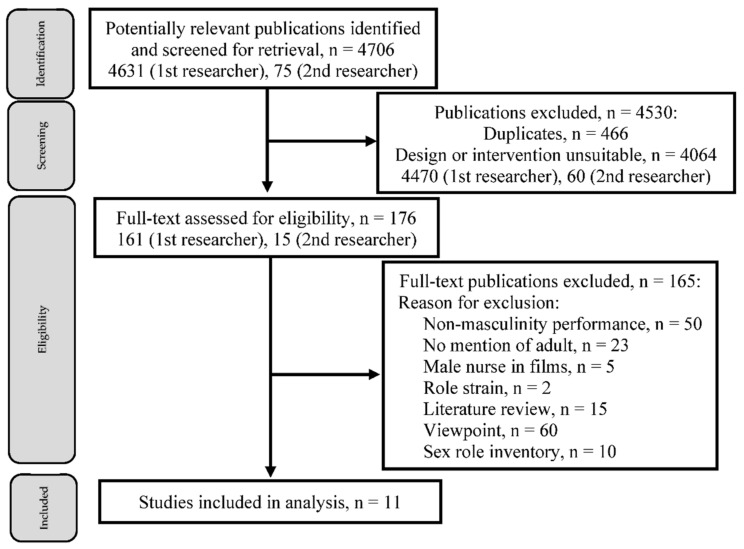
Flow diagram of search strategies, inclusion criteria, and outcome.

Initially, a total of 4706 studies were retrieved, in which 4631 studies were based on the first author’s keywords and 75 were based on the second author’s keywords. A total of 466 papers were discarded as duplicates, and 4064 papers were excluded for a lack of study design suitability. The first two authors read the titles and abstracts of the remaining 176 extracted studies to select target papers. Two additional independent researchers screened the titles and abstracts, eliminated studies not meeting the inclusion criteria, and re-evaluated the full-text versions of potentially relevant studies for eligibility. After checking titles, abstracts, and full texts, 165 papers were eliminated. A final total of 11 studies were included for analysis to address the research question of how men in nursing experience and negotiate performance masculinity in both academia and clinical practice (Figure 1).

### 3.3. Assessment of Methodological Quality

The quality of included studies was assessed using the Critical Appraisal Skills Program (CASP), a qualitative research checklist [30]. The CASP provides detailed instructions and decision rules on how to interpret the criteria used to appraise the study. This checklist contains several questions which help the reviewer to assess the rigor, credibility, and relevance of each study. All studies are critically appraised, and each study is assigned a numerical score between one and ten, where a higher score indicates a higher quality. The two studies ranked with the highest scores are used as index studies, from which the synthesized concepts are translated into other studies, and therefore, shape the analysis. The first and second authors answered the 10 CASP questions with one of three responses: yes, no, or cannot tell. The questions covered the following issues: (a) clarity of stated aim; (b) appropriateness of methodology; (c) appropriateness of design; (d) appropriateness of recruitment; (e) appropriateness of data collection; (f) consideration of the relationship between the researcher and participants; (g) consideration of ethical issues; (h) rigor of data analysis; (i) clear statement of findings; and (j) value of the research. Results of the appraisal were used to determine the general quality of the included studies and to identify potential pitfalls in the reporting that could influence the results of the review (Supplementary Materials).

### 3.4. Data Extraction and Synthesis

The first two authors read and reread each of the included studies and extracted each study’s aim, participant characteristics and setting, methodology, data collection, and analysis method (see details in Supplementary Materials).

Data synthesis for meta-ethnography differs from the traditional methods for interpreting findings derived from systematic reviews in which re-interpretation of the study findings is allowed [31]. Data synthesis for this study was specifically based on the method by Noblit and Hare [29]. Briefly, the first two authors read the 11 selected studies in full to determine the main concepts. Then they coded the results and discussion sections line-by-line to develop descriptive themes. They next compared and contrasted the descriptive themes across studies to develop analytic themes, which allowed them to generate new and more comprehensive meanings from the original data to answer the research question. They also resolved differences in coding of descriptive and analytic themes through discussion, and together they reached consensus (Table 1).

## 4. Results

### 4.1. Descriptive Characteristics of the Included Studies

The 11 studies were conducted in various countries or regions with different cultural settings, including Africa (Mauritius), the Americas (Chile and Canada), Europe (Sweden, Ireland, and UK), Australia (New Zealand and Australia), and Asia (Taiwan). Research participants included both male and female nurses in four studies [32,33,36,37]. In seven studies, only male nurses were interviewed [16,25,26,34,35,38,39]. Two of the selected studies satisfied all of the qualities on the CASP check list; eight studies scored 9 out of 10; and one study received a score of 7 out of 10 (Supplementary Materials).

### 4.2. Themes

Synthesis of the findings suggested nursing continues to be viewed as a feminine job, however it was viewed as gender-neutral in Mauritius [36]. Therefore, the theme, “Nursing could be a gender-neural occupation suitable to both genders, answered the first question, “Is nursing a gendered profession?”.

Regarding the question on masculinity of male nurses as a minority, two themes were identified: “performative masculinity” and “nursing care strategies used when providing bodywork and care”. These were derived based on the cultural views of gender and gender inequity in education and clinical practice. This situation occurs when men in nursing work cannot avoid the stigma of negative images associated with male nurses, such as being regarded as homosexual or a sexual pervert. Male nurses try to “defeminize” their behavior to maintain their masculinity when caring for both male and female patients. Synthesis of themes, categories, and findings is shown in Table 1.

#### 4.2.1. Nursing Could Be a Gendered-Neutral Occupation Suitable to Both Genders

##### Proportion of Male Nurses Varies with Country

Worldwide, the proportion of male nurses ranges from 5% to 10% in developed economies [33]. The data strongly suggest that nursing is a gendered profession dominated by females. However, an exception has been documented by Hollup [36] where 50% of employees in the nursing profession in Mauritius are men. Mauritius is classified as a middle-income country according to the UN’s Human Development index. Before its independence in 1968, most nurses enrolled at the Central Nursing School were Christians, who dominated nursing and other public sector jobs. As a patriarchal-family centered culture, nursing work that involved night duties and handling ‘impurities’ inhibited the entry of Indian women into the profession. Furthermore, in Mauritius, the equal distribution of nurses from both sexes was because of gender-segregation practice in patient care, where male nurses worked in wards with only male patients, and female nurses worked in wards with female patients [36].

Nursing in Mauritius is an interesting and contrasting case with no gender imbalance among the nursing workforce. Nursing is not seen as women’s work nor does nursing carry a low status or feminine images that would adversely impact the recruit of men enters the profession [36]. Harding reported the essential barriers that inhibited men from entering the nursing professional [40]. Moreover, nursing in Mauritius is an attractive career due to job security, good income, social mobility, employment in the public sector and possibilities for international migration [36,41].

##### Relationship between Gender and Culture

Although nursing continues to be considered a feminine job in most cultures, nursing is a viewed as a gender-neutral profession in some cultures. Nursing work in Mauritius, for example, is not perceived as feminine [33], which may be due to its culture, historical origins, and governmental policies. In addition, nurses in Mauritius provide treatments, but not patient care. Male nurses perform some tasks that only doctors perform. Male nurses often provide the initial treatment and consider themselves to be doctors’ assistants rather than “helpers.” In sum, it is advantageous to be a male nurse in Mauritius due to the following observations: (1) no stereotyping regarding gender or sexuality; (2) stable income and job security; (3) high education and social status; and (4) broader scope in duties as a physician’s assistant.

In Mauritius, nursing is not seen as women’s work, and therefore, men are more likely to enter the nursing profession. However, Hollup [36] also reported that despite a relatively balanced gender distribution between male and female nurses, sex segregation still exists in the nursing field. Due to cultural attitudes towards gender relationships and sexuality, male nurses are prohibited from working in obstetric or pediatric wards and they cannot become midwives. This form of gender segregation in Mauritius has double-edged consequences: although nursing does not have the stereotypical image of being a female occupation and male nurses are not seen as homosexual, cultural expectations regarding the behavior of heterosexuals prohibit male nurses from working in certain areas.

#### 4.2.2. Performative Masculinity

##### Avoiding the Stigma of Being a Homosexual or Sexual Deviant

Male nurses had concerns regarding being stereotyped as homosexuals in three studies [16,34,35] conducted in Ireland, Australia and New Zealand. Harding [35] indicated the hegemonic discourse of heterosexual masculinity can create the fear that homosexuality is a barrier to career progression. Some male nurses described performances that were culturally “sensitive” to the individuals in their care to resolve this predicament [35]. Heterosexuality can also be problematic for male nurses. Male nurses caring for men performed in ways ensuring identification as heterosexual, such as talking about surfing or cars [38]. While most women may see homosexuality as a feminine, non-threatening position, some may see heterosexuality as a patriarchal masculine position. Male nurses seen as “masculine” are sometimes perceived as sexual deviants [34]. When male nurses are not seen as homosexuals, some female patients may worry about sexual advances by them during physical care. These dilemmas place male nurses in a problematic position regarding the nurse–patient relationship [37].

##### Anti-Femininity or Distancing from Women

Male nurses distanced themselves from the feminine aspect of nursing by exhibiting behaviors interpreted as “masculine” [16,38]. Male nurses developed an “act” in the workplace to highlight their masculinity by adopting characteristics seen as “macho”, androgynous, physically strong, and controlling and exerting an effort to be seen as “manly”. Such distancing tactics solved conflicts between masculine identity and caring tasks seen as feminine. Harding [35] found nursing positions requiring the use of physical strength, such as psychiatric nursing, were much more tolerant and accepting of male nurses. Kumpula and Ekstrand [42] explored the experience of male nurses in psychiatric care in Sweden, which was dominated by male nurses as well as male patients. They argued that male nurses establish masculinity through physical strength, bodywork, authority and power. The interview data highlighted protection and defense as aspects of care, whereas physical strength provided status and authority. Strength was a dominant theme in two studies [32,33,39]. These representations of masculinity provide an understanding of the challenges confronted by male nurses to provide care to patients.

##### Soft Masculinity

The female-dominated culture of nursing required male nurses to exhibit “soft masculinity” [26], such as being gentle and caring while maintaining a sense of masculine identity. Male nurses used two alternative strategies: (1) conforming to the dominant heterosexual culture and (2) maintaining manhood. Understanding soft masculinity as a product of culture allowed male nurses to continually negotiate their masculine identity within a framework where the role of caring is seen as a feminine trait.

Holyoake [26] identified soft masculinity as part of a constructed identity of male nurses who have an image they wish to portray to others. This image is fashioned within nursing culture and experiences encountered in clinical practice. For example, two studies [38,39] explored male identity in psychiatric nursing. Kumpula and Ekstrand [42] suggested that soft masculinity is respected as building blocks for personal identity of male nurses in psychiatric care units. As male identities are culturally driven, they are valued in psychiatric culture. Any male nurse who does not strive to achieve these highly prized cultural signs is marginalized. Therefore, the pressure to conform is great.

#### 4.2.3. Strategies for Providing Caring and Bodywork

##### Expanded Care Work: Discursively Relabeling the Occupation

Hsu [37] created the term “expanded care” to describe niches created by male nurses in Taiwan. Male nurses identified themselves as “patient condition managers” instead of caregivers, which allowed conventional feminine nursing to be more gender-neutral or male-appropriate in the female-dominated profession of nursing. Male nurses were more likely to engage in controlling and managing nurse-patient relationships and technology-directed nursing within a “woman’s profession, and engage in “masculine situation management.” Male nurses considered themselves to be problem solvers, leaders, administrators, managers, and as technology-oriented. Male characteristics of masculinity, such as strength, aggression, independence, and ambition were also considered advantageous in difficult nursing situations [37]. Defining nurse–patient relationships as expanded care increased job positions available to male nurses in Taiwan.

##### Gender Segregation

In Mauritiu, the division of labour in patient care is sex segregated. The common practice of separating male and female patients, as well as male and female nurse, is arguably related to the perceptions of “sexualized touch.” Providing care by male nurses inevitably would involve physical touching of female patients. This may pertain to the risk of sexual abuse and molestation by the misuse of position and power.

The identity of male nurses as heterosexuals is challenged when care requires physical touch in Canada [25]. Gender segregation was typically employed when nurses of one gender avoided physical exposure or touch during bodywork on patients of the opposite sex [25,34], which was also an indication that nursing work was gender segregated. Male nurses were more cautious and vigilant about physical interactions with female patients. As fearing sexual misunderstandings and accusations, they assessed each nurse–patient situation to determine whether physical touching was safe and appropriate. Male nurses used six strategies to reduce the risk of being accused of inappropriate touching: building trust, particularly, with female patients, and maintaining formal interactions, such as shaking hands; wearing a white uniform to project a traditional image of a nurse; working in teams that included women; delegating tasks involving touching to female nurses; and minimizing body exposure by performing injections in the thigh instead of the buttock. Similar approaches were found to be used by male nurses in the study by Fisher [34] in Australia. These strategies were standard when caring for both male and female patients, which not only ensured male nurses’ safety regarding wrongful sexual accusations, but also combated the stereotypes of male nurses being homosexuals or heterosexual deviants.

## 5. Discussion

This meta-synthesis review described performative masculinity among male nurses in academic and clinical practice. Specifically, this review found that men in nursing use a number of strategies to negotiate biased gender perceptions at work that typically reflect them as being homosexual and feminine [25,26,32,33,34,35]. Male nurses attempt to eliminate these stereotypes and maintain their masculinity often by being anti-feminine, relabeling nursing tasks, joining male units, separating the care of male and female patients, using technology-directed tasks, and shaping a different type of masculinity (i.e., soft masculinity). As such, they emphasize heterosexual identity and eliminate the suspicion of homosexuality.

Research into masculinity in nursing is scant. Few nursing studies provide concrete evidence to support the assumption that the tensions of performative masculinity among men in nursing. Our findings from the 11 studies analyzed can be used to develop a male nursing model to address the gendered issues and meet the increasing demand for a male nursing workforce. Expanding and developing men in the nursing profession requires overcoming stereotypes and building an up-to-date male nursing model. The concept of hegemonic masculinity proposed by Connell [14] is internationally renowned and has been used to explain the dynamic relationships between culture and gender among males pursuing nursing careers. Stereotypes regarding gendered professions persist, which leads to gender inequality in nursing education and the high turnover rate of male nurses [34,35]. Furthermore, the reviewed studies suggest that the maintenance and application of masculinity are crucial factors for male nurses [26,34,35,37,38,39]. Although men entering the nursing profession with an attempt to eliminate occupational sex-segregation, various negative opinions and outcomes associated with gendered nursing are still ongoing [16]. The reviewed findings also indicate gender-related factors influence the nursing profession, particularly in educational institutions. Interactions among teachers, students, and peers often reflect underlying gender stereotypes or role expectations, thereby gender inequality is reinforced. Whether or not teachers automatically label men as unsuitable for the nursing profession should also be further investigated, as a fair and equal learning environment should be provided to both male and female students in nursing education, which would further improve the quality of patient care.

“Nursing like a real man” in nursing education was characterized by men’s reliance on the roles and behaviors associated with most traditional views of masculinity, such as leadership, assertiveness, and risk taking. Rather than the traditional dichotomy of masculinity and femininity, diverse features of gender identity are observed, which demonstrates the conflict between men’s masculine identity and the dominant concern in nursing education regarding caring. Gender segregation augments masculinity of male nurses by assigning different roles to males and females depending on expectations [40]. Male nursing education should be reshaped to avoid stereotyping masculinity, rejecting assumptions that male students are homogeneous, and highlighting personal traits and interests to reduce gender boundaries for caring approaches.

Currently, educational institutions in nursing have not published a well-established model of nurses that integrates males, which is a concern because an increasing number of males are entering the nursing profession. The nursing industry considers male nurses to be a solution, not only for the shortage of nurses, but also because of their traditional masculine values [37]. Given that men’s choice of entering nursing school and becoming a nurse is negatively affected by gender biases of family and society [16], an appropriate model for men in nursing could reduce the conflict between male masculinity and professional identity. Further research by nursing educators is necessary. The worldwide shortages of nurses requires an understanding of men in nursing from the standpoint of social, cultural, and historical contexts, which could provide new strategies for retaining men in the nursing profession.

### Limitations of the Review

This review had limitations. First, research exploring performative masculinity among men in nursing education and clinical practice is limited and few studies were analyzed. Second, only studies in English and Chinese were analyzed; therefore, additional aspects of male nurses’ performative behavior may not have been explored. Third, the reviewed studies published in English or Chinese were limited between 1994 and 2016. In spite of these limitations, our findings are provocative in terms of their potential application in both nursing education and professional healthcare systems.

## 6. Conclusions

This study explored the insight about the tensions of performative masculinity among male nurses, which could help address the challenges encountered by men in education, practice, and career development in nursing. Future studies must investigate the effect of performative masculinity on male nurses and establish a male nursing model based on the evidence from academic and clinical practice to meet the increasing demand for nurses. The nursing profession must decrease the tension of masculine identity associated with male nurses and a need to problematize the femininity of nursing. We suggest that academic nursing institutions and healthcare employers/administrators use multiple forms of media to propagate positive images of men in nursing, and that nursing school programs reach out to males in secondary schools and communities to recruit more men to the field of nursing. Lastly, healthcare employers and administrators should invest in male nursing students by developing mentoring programs to improve retention.

## Figures and Tables

**Table 1:** Characteristics and Descriptions of Included Studies for Meta-ethnography.

Author (Year), Country	Aim	Participants/Settings	Methodology	Data Collection	Data Analysis Method	Quality Score (CASP)	Study Code
Ayala et al. (2014) [32] **Chile**	Comprehend the socialization of male nursing students and its relationship with masculine identity.	Individual (n = 22) and group (mixed gender, n = 6) with beginning and advanced nursing students separately from a Chilean University	Grounded theory	A standardized survey; Semi-structured interviews with individuals and groups.	Conceptual integration	9/10	A
Dyck et al. (2009) [33] **Canada**	Explored the experiences of male nursing students and female nursing instructors in the context of classroom education.	Male nursing students (n = 6) who were participants in the classes and female nursing instructors (n = 6) who taught the classes.	Interpretive ethnographic study	Interviews	Structural analysis	9/10	B
Evans, J.A. (2002) [25] **Canada**	Explored the experience of male nurses and the ways in which gender relations structure different work experiences for women and men in the same profession.	Male nurses (n = 8), 20–50 years of age. 7–30 years of nursing practice. Area of nursing practice included community health nursing, mental health nursing, medical-surgical and general duty nursing. 3 leadership, 2 baccalaureate degree; 6 married, 2 lived with partner (1 with a gay man).	Qualitative	Semi-structured interviews	Thematic analysis	9/10	C
Fisher, M.J. (2009) [34] **Australia**	Report of a study examining the labor processes of male nurses in the conduct of bodywork; part of a broader study of social practices that configure masculinity through the lives of male nurses.	Male registered nurses (n = 21); 26–61 years of age; 4 identified as homosexual; 17 heterosexuals (16 married); 4 each from medical/surgical, mental health, critical care, maternal and child health, or gerontological, rehabilitation, and palliative care units. 1 military nurse.	Life history method	Semi-structured interviews	Structural analysis	10/10	D
Harding, T. (2007) [35] **New Zealand**	Examine the construct of the stereotype of male nurses as gay, and to describe how this discourse impacts on a group of New Zealand male nurses.	Male nurses (n = 17): self-identified as gay (11), or heterosexual (6). Work places included clinical nursing, education, administration, midwifery, mental health and armed forces.	Qualitative	Individual interviews	Discourse analysis	10/10	E
Hollup, O. (2014) [36] **Mauritius**	Description and analyses of how gender and cultural perceptions influenced the development of nursing in Mauritius.	Male nurses (n + 27) and female nurses (n = 20) from different grades, age, religions and ethnic backgrounds.	Qualitative	Individual interviews	Field notes	9/10	F
Holyoake, D. (2002) [26] **UK**	Explore the cultural meaning associated with male mental health nurses.	Male nurses from 2 nursing units in Birmingham and 1 nursing unit in London.	Ethnographic observation	Participant observation and in-depth interviews	Develop domain, and taxonomies (lists of related folk terms) regarding male nurse’s issues	7/10	G
Hsu, T. K. (2001) [37] **Taiwan**	Realize how male nurses redefine themselves and nursing	54 Participants: male (n = 25), female (n = 29). 28 general nurses, 4 primary supervisors, 5 top management, 2 doctors, 5 turnover male nurses. Critical care, surgical care, psychiatric care, palliative care, nursing home.	Interpretive ethnographic	Individual interviews from Male, female nurses and their leaders Field observation	Field notes	9/10	H
Huang, Y.S. & Yang, H.C. (2011) [38] **Taiwan**	Explore the masculinity experiences of four male nurses in psychiatric unit	Male nurses (n = 4) in acute psychiatric ward	Ethnography	Participant observation and in-depth interviews	Thematic analysis	9/10	I
Kumpula, E. & Ekstrand, P. (2009) [39] **Sweden**	Analyze experiences of male nurses working with male caregivers and attending to male patients in forensic psychiatry.	**Male nurses (n = 6) in psychiatric ward**	Qualitative approach	Narrative interviews	Content analysis	9/10	J
O’Connor, T. (2015) [16] **Ireland.**	Iinvestigate the gendered experiences of men choosing to be nurses	**Male nurses (n = 6) in general(adult) hospital setting ward**	Qualitative interpretive approach	Single in-depth interviews	Gender analysis	9/10	K

## Data Availability

The data that support the findings of this study are available on request from the corresponding author, H.-Y.L.

## Supplementary Materials

The following supporting information can be downloaded at: https://www.mdpi.com/article/10.3390/ijerph192214813/s1.
