# Supplementary material for: Performative Masculinity: A META-Ethnography of Experiences of Men in Academic and Clinical Nursing
Source: Int J Environ Res Public Health. 2022 Nov 10;19(22):14813. doi: 10.3390/ijerph192214813 (PMC9690486; doi:10.3390/ijerph192214813)
Supplement: Supplementary file 1 [file ijerph-19-14813-s001.zip › ijerph-1953992-supplementary.pdf]

Table S1. Quality assessment of included qualitative studies with the Critical Skills Appraisal Program.

Author(s), Date	Clear statement of aims	Appropriate methodology	Appropriate design	Appropriate recruitment strategy	Appropriate data collection	Relationship between researcher and participants considered	Ethical issues considered	Rigor of data analysis	Clear statement of findings	Is the research valuable?	Quality Score
Ayala et al., (2014)	✓	✓	✓	✓	✓		✓	✓	✓	✓	9/10
Dyck et al. (2009)	✓	✓	✓	✓	✓		✓	✓	✓	✓	9/10
Evans, J.A. (2002)	✓	✓	✓	✓	✓		✓	✓	✓	✓	9/10
Fisher, M. J. (2009)	✓	✓	✓	✓	✓	✓	✓	✓	✓	✓	10/10
Harding, T. (2007)	✓	✓	✓	✓	✓	✓	✓	✓	✓	✓	10/10
Hollup, O. (2014)	✓	✓	✓	✓	✓	✓	✓		✓	✓	9/10
Holyoake, D. (2002)	✓	✓	✓		✓			✓	✓	✓	7/10
Hsu, T. K. (2001)	✓	✓	✓	✓	✓	✓	✓		✓	✓	9/10
Huang, Y. S. & Yang, H. C. (2011)	✓	✓	✓	✓	✓		✓	✓	✓	✓	9/10
Kumpula, E. & Ekstrand, P. (2009)	✓	✓	✓	✓	✓		✓	✓	✓	✓	9/10
O'Connor, T. (2015)	✓	✓	✓	✓	✓	✓		✓	✓	✓	9/10
